# Sorry we′re open, come in we're closed: different profiles in the perceived applicability of open science practices to completed research projects

**DOI:** 10.1098/rsos.230595

**Published:** 2024-01-31

**Authors:** Jürgen Schneider

**Affiliations:** Department of Teacher and Teaching Quality, DIPF | Leibniz Institute for Research and Information in Education, Rostocker Straße 6, 60323 Frankfurt am Main, Germany

**Keywords:** open science practices, profiles, applicability, metascience

## Abstract

Open science is an increasingly important topic for research, politics and funding agencies. However, the discourse on open science is heavily influenced by certain research fields and paradigms, leading to the risk of generalizing what counts as openness to other research fields, regardless of its applicability. In our paper, we provide evidence that researchers perceive different profiles in the potential to apply open science practices to their projects, making a one-size-fits-all approach unsuitable. In a pilot study, we first systematized the breadth of open science practices. The subsequent survey study examined the perceived applicability of 13 open science practices across completed research projects in a broad variety of research disciplines. We were able to identify four different profiles in the perceived applicability of open science practices. For researchers conducting qualitative-empirical research projects, comprehensively implementing the breadth of open science practices is tendentially not feasible. Further, research projects from some disciplines tended to fit a profile with little opportunity for public participation. Yet, disciplines and research paradigms appear not to be the key factors in predicting the perceived applicability of open science practices. Our findings underscore the case for considering project-related conditions when implementing open science practices. This has implications for the establishment of policies, guidelines and standards concerning open science.

## 1. Introduction

Open science has become a significant topic of interest for political institutions and research funding agencies in recent years. This has been evidenced by the establishment of recommendations and statements promoting open science by major political institutions. Following the first United Nations (UN; [1]) open science conference, the UN Educational, Scientific and Cultural Organization (UNESCO; [2]) established recommendations on open science, emphasizing its potential to accelerate progress towards the sustainable development goals. Further, the European Commission [3–6] published statements or recommendations on open science almost on an annual basis. The rationale behind promoting open science for these stakeholders is to foster inclusion and equity, aligning with the universal human rights declaration (UN General Assembly, 1948). Furthermore, open science fosters innovation as well as practical participation and application of scientific discoveries [4]. Research funders in particular also emphasize that open science practices ensure and promote quality in the research process and output [7]. Consequently, there is a growing need to initiate a culture change towards open science in research [8].

### 1.1. One umbrella term, a wide range of aspects

Open science in itself is a broad concept. A definition, as provided on the basis of a literature review by Vicente-Saez & Martinez-Fuentes [9, p. 434] can only provide a first general idea: ‘open science is transparent and accessible knowledge that is shared and developed through collaborative networks'. This definition ties in with the Open Definition of the Open Knowledge Foundation [10,11], which recognizes the freedom to access, use, modify and share knowledge. Observing the scientific discourse, the term open science manifests itself in rather diverse dimensions [2,12]: the creation of new infrastructures, the involvement of the public in research, the discussion of metrics of scientific contribution, equal opportunities for education and knowledge, or the further development and safeguarding of research quality. These dimensions of open science concern the responsibility of different stakeholder groups of research, such as research funding organizations, research libraries, policy-making organizations or scientific societies [6]. In this paper, we focus on one of the stakeholder groups, the researchers themselves. We look at the behaviours of these researchers in the course of open science, often called open science practices. These practices include, for example, ensuring open access to research materials, research data and publications (see below for a more detailed account).

The point at which a research project should be described as ‘open’ is the subject of repeated debate. Against this background, we would like to emphasize that openness is not an all-or-nothing principle [13]. Based on the methodology and design of an individual research project, different open science practices may be relevant, shaping what open science can mean from these premises [14]. Certain open science practices are relevant for a research project, as they respond to challenges in its research domain [15], while others may not be. For example, for many qualitative-empirical research projects, preregistration of research hypotheses and design has little applicability because the hypotheses are formed during the research process (by contrast, see [16,17] for a discussion on the usefulness of preregistering qualitative-empirical research). This stance can be condensed in the statement: not all open science practices are relevant to all research projects—but to no research project is open science irrelevant.

### 1.2. Who shapes the discourse on open science?

**Figure 1.**
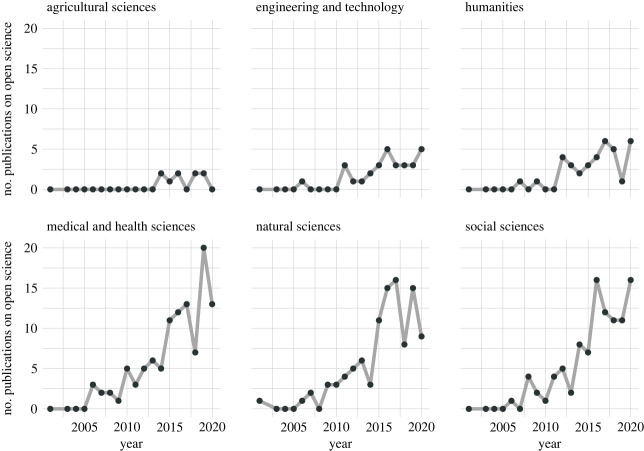
Number of publications on open science topics separated by classification of research fields from the Organization for Economic Cooperation and Development (OECD) Frascati Manual. Own analyses and illustrations based on data from the mapping review [18].

In the area of scientific publications, the discourse on open science has seen a dramatic increase in the number of contributions over the past decade. As much as open science is a topic that cuts across all research disciplines and paradigms, there are some disciplines that publish on open science more than others: data from a mapping review of open science in the literature [18] show that contributions to the discourse mainly stem from medical and health, natural and social sciences (figure 1).

There may be perfectly good reasons why these disciplines are more visible in the discourse around open science: for one, the total volume of publications differs between disciplines, and for another, the methodology and research paradigm of some disciplines are more likely to allow for openness than in others [19]. With these considerations in mind, there is substantial evidence that what we define as *openness* is more influenced by some research fields than others. This creates the risk of ‘pars pro toto’: the determination of what counts as openness in individual (more visible) research fields is generalized to many other research fields, regardless of whether or not certain open science practices are applicable in research projects within these research fields. For research fields in which certain open science practices cannot be implemented, a reverse logic is then applied occasionally: existing solutions are promoted, for which a subsequent attempt is made to find suitable problems [20]. Understandably, it can provoke a defensive stance among researchers if they feel they are being mandated to adhere to certain open science practices that are obstructive to their research projects.

We would like to illustrate this with two examples from open data practice: In 2015, 27 political science journal editors published a joint statement on ‘Data Access and Research Transparency (DA-RT)’ [21]. Through the statement, authors were required to ensure the availability of open data or otherwise notify the editor. Within only 9 days of its launch, 1173 researchers have signed a petition calling for a delay and revision of the DA-RT statement https://doi.org/10.5281/zenodo.10527770 [22]. As outlined above, the argumentation of the petition refers to the problem of prescribing practices that are not appropriate for certain research projects. This sparked a broad discussion on this topic with working groups being set up to address specific topics. The key findings of these working groups were then incorporated into a reflection paper in which alternative approaches were elaborated [14].

Similarly, in response to an increasing debate in the scientific literature and requirements from research funders as well as journals on open data, several position papers emerged from within the research community in Germany (e.g. through scientific societies). In these position papers, again, it was argued that openness in the research process is an important aspect of scientific research and that, at the same time, open data would thoroughly interfere with the research process of certain research projects [23,24]. Recent publications provide evidence that the core issue of this debate remains unsolved [25]. There is a growing recognition that research projects with specific prerequisites are systematically at a disadvantage when required to be open throughout the research process, indicating the necessity for further investigation and consideration of this issue. The consideration of different profiles in the applicability of open science practices has partly already found its way into official statements [2]. Yet, it seems important to further raise awareness because, as stated above, there is still an ongoing debate.

### 1.3. Perception of applicability by researchers

The response of researchers to the discourse surrounding open science and its potential reform messages is profoundly influenced by their *perceptions* of the applicability of the open science practices under consideration [26]. This perception serves as a guiding force for individual researchers and thus constitutes a practically relevant subject of investigation.

Researchers’ judgments of applicability are not solely contingent on evaluating the in principle applicability of open science practices [27]. In principle applicability, as an example, pertains to situations where data sharing is feasible only if a research project incorporates empirical investigation and actively engages in data collection. Beyond an evaluation of the in principle applicability, researchers' perceptions are also significantly shaped by the availability of resources in their research ecosystem [6]. These can be both internal to the researchers themselves, encompassing factors such as their understanding of archival processes and data repository usage for data sharing, and external, such as the time at their disposal for preparing data analysis code for sharing or the financial means to cover article processing charges for open access publishing in certain journals. Furthermore, these resources extend to the accessibility of user-friendly infrastructure, as for example when publicly sharing open educational resources [28]. Moreover, it is essential for the scientific community to have established standards and workflows that are readily adaptable to the specific research project at hand.

When researchers evaluate the applicability of open science practices within the context of their research project, their judgments are informed by a combination of scientific considerations (in principle) and practical considerations (resources), taking into account the unique characteristics and circumstances of their work [27].

With this paper, we aim to contribute empirical evidence to the debate that different profiles exist in the perceived applicability of open science practices and what patterns these profiles follow. Our approach involved two sequential studies: first, we conducted a pilot study to comprehensively capture the spectrum of open science practices. Second, we investigated the perceived applicability of these open science practices and their patterns, drawing on concrete research projects.

## 2. Pilot study

Among the multiple stakeholders responsible for openness in science, our attention is directed towards scientific *researchers*. Specifically, we examine the *behaviours* linked to open science practices (e.g. ‘publicly sharing research materials’ versus ‘open materials’).

The open science practices were deduced and synthesized through a top-down and bottom-up methodology, drawing upon the FOSTER taxonomy of open science (top-down; www.fosteropenscience.eu) and nine supplementary expert interviews from diverse disciplines (bottom-up). By means of the top-down and bottom-up approaches, we achieved mutual identification of blind spots to ensure a comprehensive representation of open science practices in the subsequent study. The FOSTER taxonomy was developed as part of the FOSTER Plus project, an EU-funded initiative centred on open science. The project's explicit objectives encompassed the creation of high-quality training resources, which includes the taxonomy used in this study.

### 2.1. Method

#### 2.1.1. Sample

To implement the bottom-up approach, we conducted focused interviews with nine open science experts who were recruited from an open science fellows programme in which they had served as mentors. We followed the theoretical sampling approach to ensure a diverse representation of expertise, drawing upon mentors from various disciplines such as sociology, computer science and sinology. Additionally, we deliberately included experts from different research paradigms, including qualitative, quantitative, mixed methods and theoretical.

#### 2.1.2. Procedure and materials

After a short introduction to the study, interviewees were given a narrative prompt that directed them to retrospectively reflect on open science practices within their field. Specifically, they were asked to recall a recently completed research project and to consider various aspects of open science that they deemed relevant, accompanied by specific examples of how such practices could be exemplified in research projects: ‘please recall one of your most recently completed research projects. If you think about the entire project span, from the first idea to the completion of the project: which aspects of open science do you consider to be relevant and how can they be exemplarily implemented in research projects?’

After the interviewee signalled the end of the narration, the interviewer then posed additional follow-up questions. These inquired about further open science practices relevant in his/her field, by asking ‘are there other aspects of open science that you consider to be relevant in your research projects (i.e. potentially others as well)? If so, how could these be implemented?’.

Finally, the interviewer posed follow-up questions to clarify any practice referenced by the interviewee that remained ambiguous: ‘you talked about … before. Can you make this aspect a little clearer with an example? How would the aspect be implemented?’

The interview script and other materials can be openly accessed on https://doi.org/10.5281/zenodo.7886776 [29,30].

#### 2.1.3. Analyses

Following the focused interview, two trained coders transcribed and segmented the interview material to identify each open science practice mentioned. This resulted in 253 distinct segments that addressed various aspects of open science. Among these segments, 35 were excluded because they did not satisfy our predetermined inclusion criteria. Specifically, these segments either did not pertain to researchers themselves (e.g. infrastructure requirements) or did not concern specific behaviour (e.g. attitudes or motivation). The remaining 218 segments were subsequently coded via qualitative content analysis that occurred in two stages. First, the coders formulated general descriptions of the practices by abstracting the behaviours named by the interviewees. Each description was named using characteristic words from the interviews. Categories with similar practices were grouped together and collapsed into one category. Second, these practices were compared and synthesized with the FOSTER taxonomy. Similar practices were grouped together and collapsed into one category. Names for these categories were updated based on both the interview material as well as the FOSTER taxonomy. Both trained coders segmented and coded the entire material. Throughout the segmenting and coding process, disagreements were resolved through discussion.

### 2.2. Results

The top-down (taxonomy) and bottom-up (interviews) approach resulted in the definition of 13 distinct open science practices. Based on the Open Science Training Handbook [31], we established definitions for each of the 13 open science practices. To render them as items that could be used in the subsequent study, we created concise headings, accompanied by clear explanations and terms known from the open science movement (table 1).

**Table 1.** The 13 open science practices that resulted from the top-down (taxonomy) and bottom-up (interviews) approach and their count in the interviews.

open science practice	count	example from interview
**involving the non-academic public in the research process.** The non-academic public is involved in the process of scientific research—whether in community-driven research or global investigations. Citizens do scientific work—often working together with experts or scientific institutions. They support the generation of relevant research questions, the collection, analysis or description of research data and make a valuable contribution to science. ‘Citizen science’	15	‘but what I find much more exciting is […] the opening of science for the people who are involved in science in some way, also and then subsequently for society. That is, there is now a lot of talk about citizen science, or about participation […]. That has always bothered me […] we somehow try to generate empowerment, we try to change things in society, but how we treat our research subjects and how we deal with their data, and how we give them credit for what they have done in our research, there is still a lot of room for improvement’
**publicly sharing project plans to encourage feedback and collaboration.** Researchers make their project plans publicly available at an early stage (e.g. on social media, websites) to optimize the study design through feedback and to encourage collaboration. ‘Open collaboration’	5	‘so there is this idea that you share something like that […] that is, you already share project proposals. So, I once worked on a project with […], who once submitted a large proposal, I think, and also made it quite public […] Of course, there is this idea that you discuss, okay, if you share everything now, then someone else can maybe push it further’
**preregistering study plans.** Researchers submit important information about their study (for example: research rationale, hypotheses, design and analytic strategy) to a public registry before beginning the study. ‘Preregistration’	3	‘what we don't do, […] is this preregistration stuff. Which often also has to do with the fact that most of the work that I do at least, are not really so much deductive, in nature. […] A lot of this preregistration stuff is rather, let's say, relevant to work that proceeds deductively, where there are clear hypotheses that then have to be tested’
**publicly sharing the methodology of the research process.** Researchers describe methods, procedures and instruments that are used in the process of knowledge generation and make them publicly available*. ‘*Open methodology*’*	26	‘so now you can also look […] at the methods, at open methods. You can think about: ‘what dimensions of my method can I open up?’ And we have just started to experiment, […] I do a lot of discourse-analytical things, that is, where we, for example, annotate, categorize, cluster documents, cluster terms, and so on, […] and there you can, for example, very well something like annotation scheme, category scheme, codebooks, and so on, right? You can publish all that. And that shows how the process of knowledge generation has come about over many documents, for example, or interviews’
**using open file formats and research software.** Researchers use software (for analysis, simulation, visualization, etc.) as well as file formats that grant permission to access, re-use and redistribute material with few or no restrictions. ‘Open file formats and research software’	4	‘so, all this open source software stuff, […] the only software I use is qualitative data analysis systems, so I use MAXQDA, what else is there, two others, ATLAS.ti and NVivo, […] none of them is open source, […] they are pretty much for-profit’
**publicly sharing research materials.** Researchers share research materials, for example, biological and geological samples, instruments for measurement or stimuli used in the study. ‘Open materials’	3	‘there were also music documents and I think […] learning documents that should also be put openly in our project’
**publicly sharing data analyses.** Researchers make the procedure of the data analyses and their scripts (code) publicly available so that others are able to reach the same results as are claimed in scientific outputs. ‘Open code/open script’	8	‘if you do a small replication, you can do it based on the data that we have created and based maybe on the analysis scripts that we have created, just to see if the calculations are correct, right? So, the, so to speak, the small variant, which I think can be so very powerful. In general, for quality assurance in science, I mean’
**publicly sharing research data.** Researchers publicly provide the data generated in the research process free of cost and accessible so that if can be used, reused and distributed provided that the data source is attributed. ‘Open data’	62	‘but what you can also do […] is that I can of course also anonymize my data. Because this is structured data, where you could theoretically also say, I anonymize this dataset, so that, for example, instead of the names, you only have numbers, and I pull out this dataset, so to speak, anonymize it, and then also put it online’
**generating open educational resources.** Researchers produce and release teaching, learning and research materials in any medium that reside in the public domain or have been released under an open license that permits no-cost access, use, adaptation and redistribution by others with no or limited restrictions. Open educational resources include full courses, course materials, modules, textbooks, streaming videos, tests, images, software, and any other tools, materials or techniques used to support access to knowledge. ‘Open educational resources’	5	‘theoretically, you could also open it up in such a way that I now publish a teaching concept or something. […] But the other thing is that this is so special, […] it's just always a bit of a question for me: ‘okay, who uses this?’ […] but for example next semester I have a course on […] and I could imagine putting a whole curriculum online. Right? And learning resources and so on’
**deciding for openness in the peer review process.** Researchers opt for some kind of openness in the peer review process, including making reviewer or author identities open, publishing review reports or enabling a broader community to participate in the process. ‘Open peer review’	7	‘we just publish it as a special issue, […] where the reviews, that is, where the reviewers are named. Where the reviewers know who the authors are, and the authors and reviewers can decide whether the reviews will also be published, for example, on the paper, right?’
**publishing open access.** Researchers publish their research paper online, free of cost with free reusability regarding copyright restrictions. This involves any form of open access (preprints, gold and hybrid open access, etc.). ‘Open access’	39	‘although it's not always the case that I would only prioritize pure open access journals. So I know with the last project, it was about that, we looked: Which journals are there covering the topic at all? But that was the case because I was supposed to publish in Q1, that is, in high-ranking journals, and it was difficult with open access’
**providing open source code of software.** Researchers make source code for a piece of software that was developed in the research process publicly available, along with an open source license permitting reuse, adaptation and further distribution. ‘Open source’	10	‘that computer science has always been very open, so when I started doing my PhD, it was always clear that if it was open source, that we would always develop open source’
**communicating research results to non-academics.** Researchers use appropriate skills, media, activities, and dialogue to produce one or more of the following personal responses to science: awareness, enjoyment, interest, opinions, understanding. Science communication may involve science practitioners, mediators and other members of the general public, either peer-to-peer or between groups. ‘Science communication’	31	‘so, we have a website, which we maintain quite well, […] where we try to always keep news and such up to date, […] And then we have now also set up a Twitter account, […]. And what you see here now is on the one hand, what we have done in terms of research, so what were the research questions that have kept us busy […] Then here are all the publications linked to it again’

## 3. Main study

In the second study, we investigated whether different profiles exist concerning the perceived applicability of open science practices and what patterns these profiles follow in research projects across disciplines. Descriptions of all research materials and data are publicly available in the data note publication and associated repository [29].

### 3.1. Method

#### 3.1.1. Sample

To evaluate the perceived applicability of open science practices, we requested researchers to provide their estimates based on their prior research projects. We followed the Organization for Economic Cooperation and Development (OECD) Frascati Manual [32] classification of research fields, to ensure a diverse sample across research disciplines. Our objective was to recruit *n* = 50 participants for each ‘broad classification’ group, encompassing natural sciences, engineering and technology, medical and health sciences, agricultural and veterinary sciences, social sciences, humanities and the arts, for a total sample of *N* = 300, constrained by financial resources. Owing to a natural delay in closing groups when they were complete (some participants still responding), two groups had slightly more participants than intended. In addition, the narrow focus of agricultural and veterinary sciences made it challenging to enroll a full sample. This resulted in *N* = 295 participants, with *n* = 52 from natural sciences, *n* = 50 from engineering and technology, *n* = 50 from medical and health sciences, *n* = 42 from agricultural and veterinary sciences, *n* = 51 from social sciences and *n* = 50 from humanities and the arts following the classifications of the Frascati manual. Participants were recruited through the online access panel provider prolific.co. The study description for the participants in prolific merely indicated that the study addressed practices in research projects while avoiding emphasis on open science to reduce selection bias in the sample. Only participants who confirmed that they worked in scientific research and were fluent in English (in order to understand the questionnaire) were admitted to the recruitment process. Accordingly, the largest proportion of participants came from the USA (*n* = 91) or the UK (*n* = 62). The proportion of women was 56%.

#### 3.1.2. Design

We applied a cross-sectional survey using the web-based survey tool formr [33]. Rather than asking about time periods (e.g. the last year), the survey focused on the applicability of open science practices in concrete research projects. To enable informed assessments and ensure ecological validity, we asked participants to evaluate the perceived applicability of open science practices in research projects that had been completed, thereby allowing them to draw upon their experiences from all phases of the project. See the codebook file [34] in Schneider [29] for further details on the data collection.

#### 3.1.3. Measures and procedure

After providing informed consent, participants were instructed to select a completed research project for which they would answer the subsequent questions [29]. The first question, which was also used as a filter to regulate sample sizes, asked participants ‘to which discipline is this research project most closely related?’ Response options consisted of a drop-down menu of all 42 second-level classifications from the OECD's Frascati Manual [32]. These second-level classifications were used as response options to allow participants to specifically, and thus more easily, assign their research project to the classifications. Owing to a large number of response options of the second-level classifications, we expected resulting small cell counts and therefore planned to use the broader classification group of each participant's discipline for further analysis.

Next, we administered an attention check that consisted of one item: ‘please read the following scenario briefly and answer a question about it:

a famine has broken out in your village. You and some others have been chosen to leave the village and search for food. It begins to rain heavily and soon there will be flooding. Participants in studies like this are sometimes not very attentive. We have included this question here to check if you have actually read the scenario. If you read this, leave the following question unanswered just click next.

According to the scenario, would it be appropriate to take the raft and leave the others behind?’ The answer options included a seven-point Likert scale with the anchors ‘absolutely no’ and ‘absolutely yes’. To pass the attention check, participants should not have marked anything on the 7-point Likert scale, resulting in a missing value for this item. Out of the participants who were eligible to participate, 20 failed the attention check, were skipped to the end of the survey and were excluded from further analysis [29].

On the following page, participants who successfully passed the attention check received a reiterated instruction to reflect on a completed research project. Additionally, we emphasized that the study's primary focus was on the potential applicability of practices rather than the actual implementation of these practices in the project: ‘when answering the items on the next page, please think of a research project of yours that you have already completed. Regardless of whether you *actually applied* the practices in this research project: which of the practices would have been *potentially applicable*, given all the characteristics and circumstances of the project? This includes both scientific, and practical considerations in conducting the study’.

Subsequently, we asked participants about their assessment of the applicability of 13 open science practices for their research project: ‘to what extent are the following behaviours applicable in your research project?’ This was then followed up by the items as displayed in table 1. The response format was that of a Likert scale with four response options ranging from ‘not applicable at all’ to ‘highly applicable’.

Lastly, we assessed the research paradigm the project was situated in: ‘research paradigm. What was the project's primary research interest and design?’ with the single choice answer categories ‘mainly qualitative empirical’ (*n* = 60), ‘mainly quantitative empirical’ (*n* = 104), ‘explicitly mixed-methodological (equally qualitative and quantitative empirical)’ (*n* = 119) and ‘non-empirical’ (*n* = 12). The authors decided in favour of ideal-typical distinctions between research paradigms. We recognize that these do not necessarily exist in this unambiguous form in the reality of research practice. However, in order to gain a first impression of differences between the paradigms, the participants were given the opportunity to categorize their research projects into one of the categories.

#### 3.1.4. Statistical analyses

We used the R package tidyLPA [35] to identify latent subpopulations based on the perceived applicability of 13 open science practices. Our analysis included the estimation and comparison of one to eight classes. To determine the optimal number of profiles, we evaluated both the Bayesian information criterion (BIC), which is typically considered the most accurate index [36], and entropy, which assesses the ability of a mixture model to return well-separated profiles [37]. We then employed the research paradigm and discipline of the research project as dummy coded predictors to the profile probabilities. In the case of significant results, we additionally computed Tukey-adjusted contrasts within the dummy coded predictor.

We preregistered the sample size, design and data analysis of the study on https://doi.org/10.17605/OSF.IO/297Y8 [38]. Initially, our preregistration involved using latent class analysis. However, as the indicators would be treated as categorical, this approach was not entirely suitable. Therefore, we switched to using latent profile analysis (LPA) instead, which better accommodated our study design. For transparency reasons, we included the originally planned data analysis alongside the updated data analysis in the reproducible documentation of analysis (RDA): https://doi.org/10.5281/zenodo.7886776 [30].

### 3.2. Results

Looking at the overall descriptive data, we see that, from the researchers' point of view, open methodology can be implemented most frequently (table 2). This is also where there is the least variance, i.e. differences between the research projects. By contrast, the provision of open source code is, arguably hardly surprising, the least widespread. Moreover, this is also where there is the greatest variance between the researchers’ assessments.

**Table 2.** Mean and standard deviation of agreement on the applicability of open science practices*.* (Note: the scale on all items ranged from one to four.)

open science practice	mean	s.d.
open methodology	3.214	0.921
open access publication	3.088	1.016
open data	3.051	1.014
open materials	3.014	1.000
science communication	2.980	0.940
open code	2.888	1.029
open peer review	2.871	1.039
public project plan	2.837	1.021
open software	2.810	1.019
open educational resource	2.512	1.112
preregistration	2.508	1.100
citizen science	2.478	1.169
open source	2.281	1.183

#### 3.2.1. Latent profile analysis

**Figure 2.**
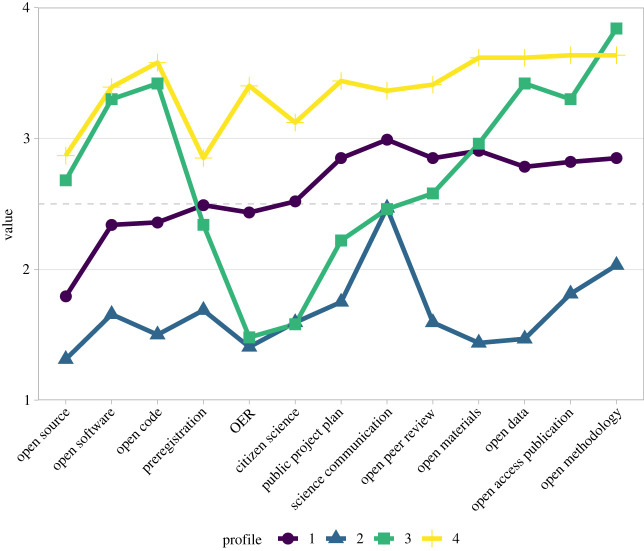
Structure of the four latent profiles over the 13 open science practices and their mean scores on the 1–4 Likert scale. OER, open educational resources.

The LPA results indicate a four or five profile solution: The BIC fit index is lowest for the solutions with four and five classes (for details, see link to RDA). For the five class solution, however, the entropy falls under the threshold of 0.80. We, therefore, decided for the four class solution with an acceptable entropy (0.817) (figure 2).

##### 3.2.1.1. Profile 4: fully open (36%)

According to the researchers' evaluations, the research projects in this profile demonstrate a high degree of consistency in their ability to apply a wide range of open science practices. While this group shows the lowest values in providing open source software and preregistration, the values are still well within the range of agreement.

##### 3.2.1.2. Profile 3: open with low public participation (17%)

This name reflects the fact that research projects in this group are perceived as showing particular strengths in large parts of the open science practices, while also being limited in the creation of open educational resources and implementation of citizen science. In other practices such as science communication, research projects from this group show mixed results.

##### 3.2.1.3. Profile 1: potential for openness (36%)

Research projects in this group exhibit potential but not a strong tendency toward openness in nearly all open science practices. Except for sharing open source code, probably owing to the fact that source code is not always an outcome of research projects. This group perceives slightly higher levels of applicability in some open science practices than in others. Notably, well-established practices that are widely discussed in the open science discourse, such as open access publication, open data, and open methodology [18], show a greater prevalence of perceived applicability in this group.

##### 3.2.1.4. Profile 2: maybe science communication (11%)

In this group of research projects, there is a low probability of openness across most open science practices, with one notable exception: science communication. A significant difference from the other groups is that even the practices with the highest prevalence across all groups, such as open methodology and open access publication, tend to be perceived as inapplicable.

#### 3.2.2. Predicting conditional probabilities

In our analysis of predicting profile probabilities, we observed that the explained variance was not substantial (around 6% at most). Our findings suggest that the research paradigm and discipline may not be the most relevant predictors, based on our operationalization. We nevertheless identified predictors that elicited a substantial change in conditional probability in three of the four profiles (table 3).

**Table 3.** Linear regression models to predict conditional probabilities of class membership. (Note: we report the coefficient, standard error and *p*-value for each predictor. Reference categories: mixed methods (paradigm); agricultural and veterinary sciences (discipline). **p < 0.01.)

	dependent variable			
	***p*[profile 1]**	***p*[profile 2]**	***p*[profile 3]**	***p*[profile 4]**
*paradigm*				
none	0.041	0.221	−0.080	−0.128
	(0.303)	(0.307)	(0.301)	(0.304)
	*p* = 0.893	*p* = 0.472	*p* = 0.792	*p* = 0.674
			
qualitative	0.446	0.146	−0.202	−0.369
	(0.159)	(0.161)	(0.158)	(0.159)
	*p* = 0.006	*p* = 0.366	*p* = 0.201	*p* = 0.022
			
quantitative	−0.039	0.068	0.172	−0.141
	(0.133)	(0.135)	(0.132)	(0.134)
	*p* = 0.768	*p* = 0.616	*p* = 0.196	*p* = 0.293
*discipline*				
engineering and technology	−0.295	0.226	0.253	−0.067
	(0.208)	(0.210)	(0.206)	(0.208)
	*p* = 0.157	*p* = 0.284	*p* = 0.221	*p* = 0.748
			
humanities and the arts	−0.226	−0.073	0.439	−0.074
	(0.211)	(0.213)	(0.209)	(0.211)
	*p* = 0.286	*p* = 0.735	*p* = 0.037	*p* = 0.728
			
medical and health sciences	−0.123	−0.185	0.118	0.152
	(0.208)	(0.211)	(0.206)	(0.209)
	*p* = 0.556	*p* = 0.382	*p* = 0.569	*p* = 0.467
			
natural sciences	−0.178	−0.149	0.643	−0.225
	(0.206)	(0.208)	(0.204)	(0.206)
	*p* = 0.389	*p* = 0.476	*p* = 0.002	*p* = 0.277
			
social sciences	−0.318	−0.054	0.158	0.219
	(0.207)	(0.210)	(0.205)	(0.207)
	*p* = 0.126	*p* = 0.799	*p* = 0.442	*p* = 0.293
*R*^2^	0.045	0.021	0.061	0.040
adjusted *R*^2^	0.018	−0.007	0.034	0.013
residual s.e. (d.f. = 286)	0.991	1.003	0.983	0.993
*F* statistic (d.f. = 8; 286)	1.676	0.759	2.311**	1.493

In the case of profile 1, which indicates a potential for openness, our analysis revealed higher probabilities for research projects following the qualitative paradigm. Specifically, we found that the probability of belonging to this profile is 45% higher for qualitative projects compared to mixed-methods projects, and 49% higher compared to quantitative projects.

The probability of affiliation with profile 3, characterized as ‘open with low public participation’, increased by 64% for natural science research projects in comparison to agricultural research projects. Furthermore, research projects from humanities and the arts demonstrated a 43% higher probability of belonging to profile 3, compared to agricultural research projects as well.

Finally, qualitative-empirical research projects had a 37% lower probability of belonging to profile 4 (fully open), compared to mixed-methods projects.

## 4. Discussion

The objective of our study was to provide empirical evidence regarding the question if different profiles of researchers' perceived applicability of open science practices exist. Based on researchers' assessments from 295 research projects, we found that different profiles do exist. We identified four profiles that ranged from being fully open to rather closed, with researchers conducting qualitative-empirical research projects perceiving a lower probability of being fully open, but still exhibiting potential for openness (profile 1). As argued at the beginning regarding the examples of open data, there may be several constraints (e.g. data protection) that make it appropriate and important not to be fully open.

In the formulation of open science reforms and policies, it is natural to involve authors with extensive experience in open science, typically from research fields that readily embrace and implement open science practices (‘fully open’ profile). Our paper substantiates the need of engaging researchers who have direct experience in projects facing barriers related to adhering to conventional definitions of openness. The distinct profiles introduced in our study serve as indicators, shedding light on the diverse barriers that researchers may perceive in the research process and the open science practices they feel comfortable to feasibly embrace.

While the immediate recognizability or ease of characterization of these profiles may be subject to debate, their primary function lies in acting as a heuristic to remind the research community about the perceived spectrum of openness possibilities throughout the research process. These may rely on perceived (possibly practical) aspects and not necessarily factors that influence the in principle applicability of open science practices; nevertheless, these perceptions guide decision-making in the research process. Hence, our analysis underscores the necessity for a finer and more detailed examination of the applicability of open science practices, surpassing broad discipline categories and research paradigms. The evidence from our findings emphasizes the existence of different patterns in the adoption of open science practices according to the researchers' reality, demanding due recognition. Consequently, the implications underscore the importance of tailored approaches and investigations of barriers as well as facilitators, when addressing open science within different research contexts. These barriers and facilitators (e.g. the accessibility of user-friendly infrastructure) may vary strongly between contexts, such as countries, or research institutes. In particular, researchers who are already disadvantaged may find it difficult to implement open science practices that would in principle be applicable but cannot be realized owing to a lack of resources. As many research funders and journals already require a range of open science practices, these researchers working in precarious circumstances are further disadvantaged. By acknowledging the distinctions in the perceived applicability of open science practices and embracing a more nuanced understanding, the research community can develop more effective and contextually appropriate strategies to promote openness and foster a culture of open science.

### 4.1. Limitations and further research

In our study, our primary focus centres on researchers' perceptions of the applicability of open science practices. As a result, our assessments consider all relevant aspects within research projects, encompassing both scientific and practical dimensions. While this approach enhances the ecological validity of our evaluation, we acknowledge that these judgments may not always align with the epistemological or in principle applicability of these practices. Within our data collection, we face a challenge in differentiating between the sources of these judgments. We designed our survey questions to encourage participants to reflect on both the practical and in principle aspects, but we cannot definitively determine whether their judgments are primarily driven by practical considerations or by in principle reasons. A more direct investigation of the inherent characteristics of research projects themselves, without assessments through the researchers' lens, would be required to disentangle these sources of judgment. Alternatively, a more detailed analysis of researchers' reasoning processes when assessing the applicability of each open science practice could shed further light on the specific challenges related to applicability.

Furthermore, a discussion of the statistical approach is warranted. In LPA, as an exploratory approach, researcher degrees of freedom could exert an influence on both the number and pattern of the resulting profiles. To address these concerns, we implemented specific measures at two levels to mitigate bias. Firstly, achieving a balanced sample was prioritized. This facilitated the incorporation of diverse disciplines into the evaluation, accounting for their unique features that would have otherwise remained unnoticed during the data analysis. Secondly, recognizing the potential influence of researcher degrees of freedom on data analysis results, we adhered to standard procedures and used established cut-off values in the application of the exploratory approach. Owing to the initial preregistration for a latent class analysis and subsequent updating to an LPA, both analyses were performed to ensure transparency. This multiple data analysis approach provided a preliminary indication of the robustness of the results with respect to the chosen analytical method. Irrespective of the analytical method used, the cluster analysis yielded consistent outcomes to the LPA: one cluster resembling profile 4 (fully open), another cluster resembling profile 2 (maybe science communication), and a third cluster combining profile 1 (potential for openness) and profile 3 (open with low public participation) into a unified class.

Of the four profiles, the ‘maybe science communication’ profile encounters the most barriers to openness. Acknowledging our study's limitations, we cannot determine the reasons behind the limited openness of these projects. Possible barriers could include the need for subject protection, the risk of dual use, inadequate infrastructure to support openness, or limitations by design. Yet, it is noteworthy that this profile constitutes the smallest share of research projects (11%) of the research landscape compared to the other profiles. Barriers to the application of open science practices, which become relevant for these research projects, are already well-studied in some areas. One example is the implementation of open data, which continues to suffer from the lack of well-established research data centres on the one hand, and from poorly established standards and resources on the other. According to data from the State of Open Data Survey [39], 54% of the participants reported experiencing at least one of the following uncertainties: (i) uncertainty regarding the accessibility of data according to FAIR principles [40], (ii) uncertainty regarding the rights to share data, (iii) uncertainty regarding copyright and licensing issues, or (iv) uncertainty regarding the selection of a research data centre. Regarding challenges with open data, the European Commission's [5] open science monitor further reveals that only 27% of the respondents reported receiving adequate training and education on making research data available. Hence, establishing and disseminating standards and resources for researchers to implement open science practices remains a key challenge. Thus far, the responsibility for this has been assigned to scientific societies of the respective disciplines. While this differentiation is advisable, our study has revealed significant variability in the applicability of open science practices even within the same discipline. As we were unable to identify the factors that contribute to this variability, this requires further investigation.

As evident from the results section, the operationalization of the research paradigm and discipline in the study does not demonstrate significant predictive power over the conditional probabilities. For future research, we propose addressing the two dimensions of research paradigms and research fields individually, to facilitate a more in-depth investigation of the topic. One potential approach could involve obtaining a higher sample size to enable a more fine-grained distinction of disciplines, followed by an investigation to identify any similar profiles among these disciplines. Moreover, the incorporation of an open text format item to assess the research paradigm could enhance ecological validity. This format would be beneficial for capturing the nuances particularly within the qualitative research paradigm, which encompasses a diverse range of methodological approaches that may exhibit limited commonalities and, consequently, reduced predictive power.

## 5. Conclusion

Taken together, we understand these results to support the notion: not all open science practices are perceived relevant to all research projects—but to no research project is open science irrelevant. Our study provides empirical evidence that different profiles exist in the implementation of open science practices, taking researchers' perspective into account. While for researchers in some projects being fully open is feasible, others can only focus on science communication, and many fall somewhere in between with their own strengths in certain areas of openness. However, even researchers in projects that are not fully open see the potential to open up in certain aspects, such as science communication. On the other hand, some researchers show high perceived applicability of openness of their research, but struggle to engage the public. To advance open science, we need to remove the perceived barriers that prevent the application of open science practices, while also accepting the non-application of open science practices when there is no other feasible option.

## Data Availability

The article's supporting data and digital research materials can be accessed on https://doi.org/10.5281/zenodo.7886776 [30]. There is also a data note publication available with a more detailed description of the dataset [29].
